# The Tail Domain Is Essential but the Head Domain Dispensable for *C*. *elegans* Intermediate Filament IFA-2 Function

**DOI:** 10.1371/journal.pone.0119282

**Published:** 2015-03-05

**Authors:** Kyle Williams, Kristen Williams, Hallie M. Baucher, John Plenefisch

**Affiliations:** Department of Biological Sciences, The University of Toledo, Toledo, Ohio, United States of America; University of Vienna, Max F. Perutz Laboratories, AUSTRIA

## Abstract

The intermediate filament protein IFA-2 is essential for the structural integrity of the *Caenorhabditis elegans* epidermis. It is one of the major components of the fibrous organelle, an epidermal structure comprised of apical and basal hemidesmosomes linked by cytoplasmic intermediate filaments that serve to transmit force from the muscle to the cuticle. Mutations of IFA-2 result in epidermal fragility and separation of the apical and basal epidermal surfaces during postembryonic growth. An IFA-2 lacking the head domain fully rescues the IFA-2 null mutant, whereas an IFA-2 lacking the tail domain cannot. Conversely, an isolated IFA-2 head was able to localize to fibrous organelles whereas the tail was not. Taken together these results suggest that the head domain contains redundant signals for IF localization, whereas non-redundant essential functions map to the IFA-2, tail, although the tail is unlikely to be directly involved in fibrous organelle localization.

## Introduction

Intermediate filaments (IFs) form stress resistant cytoskeletal networks that reinforce cell structure and transfer force from individual cells to neighboring cells or extra-cellular matrix via junctional complexes, contributing to tissue integrity [1,2,3]. They can also interact with signaling molecules and potentially regulate signaling pathways [3,4]. Congenital or acquired disruptions of lamins, cytoplasmic IFs, and IF-containing junctions are associated with tissue fragility, a variety of human diseases and developmental abnormalities [1,5]. Mutations that disrupt cytoplasmic IFs, desmosomes or hemidesmosomes have been shown to result in developmental abnormalities in experimental model systems, including *Caenorhabditis elegans* where mutation or RNAi disruptions of epidermal IFs result in tissue separation, failure of embryonic elongation and muscle collapse from the body wall [6,7,8].

Intermediate filaments are polymers of individual IF proteins that share a conserved structure, consisting of an extended alpha helical rod domain flanked by globular N terminal head and C terminal tail domains [2]. The rod domain is essential for the direct interactions between IF proteins leading to polymerization into filaments, and is highly conserved between all IFs including the nuclear lamins [9]. The globular head and tail domains are more diverse, varying in sequence and size between different IF proteins. Biochemical and rotary EM shadowing studies have shown that individual IF proteins form parallel dimers through the interaction of their rod domains; these are homodimers or heterodimers depending on the specific IFs involved [2]. The dimers form anti-parallel tetramers and these assemble into higher order structures and ultimately the mature filaments. Rotary EM shadowing of in-vitro assembly intermediates and mature filaments of cytoplasmic IF proteins from the nematode *Ascarais suum*, shows that the globular head and tail domains project orthogonal to the long axis of the rod, thus in the mature filament they provide a significant portion of the exposed IF surface [10]. While evidence from mammalian and *C*. *elegans* systems shows that the globular domains contribute to IF assembly, and their phosphorylation can modulate IF assembly[11,12], their exposed position suggests they are likely to be the principal mediators of interactions between IFs and other intracellular complexes, including hemidesmosomes. This exposed positioning, coupled with the diversity of these domains, makes it likely that the head and tail will determine most of the distinctive differences and cell-type specific functions of IFs found in different cell types.

In *C*. *elegans*, the cytoplasmic IF proteins IFA-2, IFA-3, IFB-1 and IFC-1 are expressed in the epidermis, where they contribute to the mechanical connections that transmit the force of skeletal muscle contraction to the cuticle [6,7,8,13,14]. The skeletal muscles lie in four longitudinal bands immediately below a basal lamina separating them from the epidermis. The muscles form integrin-mediated attachments to this basal lamina [15,16] At the region of muscle contact, the epidermis assembles specialized matrix attachment complexes, the fibrous organelles (FOs), consisting of basal and apical hemidesmosomes linked by the cytoplasmic IFs that serve to transmit force from the basal lamina to the overlying cuticle [15,17]. IFA-2 and IFB-1 have been shown to localize to the FOs, and mutant or RNAi disruption of IFA-2, IFA-3, IFB-1 or IFC-1 result in a fragile epidermis associated with muscle collapse away from the cuticle [6,7,8,14] that is similar to that of mutations that disrupt the structural integrity or assembly of hemidesmosome components of the FO [e.g. [18,19,20]. IFA-3 and IFB-1 are required in the embryo, whereas IFA-2 is needed in juveniles [6,7,8,13]. In the case of the *C*. *elegans* epidermal IFs, IFB-1 has been shown to heterodimerize with both IFA-2 and IFA-3 [13].

FOs form in the embryo in response to interactions between the muscle cells and the epidermis. Epidermal cytoplasmic IFs, as recognized by antibodies, initially accumulate in the dorsal and ventral epidermis (270 min), become organized into patches as the muscle cells migrate into their final positions (310 min-two fold stage), and organize into FO complexes that align with the cuticular annuli and are restricted to regions of muscle contact by the two-fold stage [21,22]. Contact with muscle cells is required for organizing the cytoplasmic IFs, they are absent from the epidermis when individual muscle cells or groups of cells are ablated or where neuronal bundles interpose between muscle and epidermis [22]. No single protein has been demonstrated to be absolutely required for the initial assembly of IFs or their recruitment to nascent FOs, one possibility being that the system contains redundancy. Myotactin, the matrix receptor located in the basal hemidesmosome may contribute to this process, however, since organization of the cytoplasmic IFs and their spatial restriction precedes the recruitment of myotactin to the complexes, myotactin is probably not directly involved in initial FO assembly [22]. The spectraplakin VAB-10 may play a role in localizing cytoplasmic IFs to the FOs, the loss of function phenotype of *vab-10* shows a disruption of cytoplasmic IF localization, however FOs are still initially formed and localized [23]. SUMOylation of the tail of *C*. *elegans* IFB-1 modulates its incorporation into the FOs, and it is predicted that phosphorylation of the IFA-3 tail may have similar effects [12,24]. It is unclear if these modifications are affecting IF assembly, their recruitment to the FOs, or both. It is also unclear which IF domains are mediating the interaction between the IFs and the hemidesmosome portion of the FO, although we would predict that the head and/or tail play important roles.

To assay contribution of the head and tail domains to IF function, we specifically generated IFA-2 variants lacking one or both domains, and tested whether the variants localized to the FOs and if they can substitute for full-length IFA-2 in animals lacking endogenous IFA-2. We show that IFA-2 deleted for the head domain localizes properly and functionally substitutes for wild-type IFA-2. In contrast, IFA-2 specifically deleted for the tail domain, although retaining the ability to localize properly, cannot functionally substitute for wild-type IFA-2. In addition we show that the head domain, but not tail domain of IFA-2 can localize to FOs in the absence of the rod domain. These observations suggest that neither the head nor tail domains of IFA-2 are essential for assembly of IFA-2 into filaments, that the tail but not the head domain contributes IFA-2 specific function, and that the head domain’s function likely contributes to localizing the cytoplasmic IFs to the FOs by direct interactions.

## Materials and Methods

### Worm culture and strains

Standard 20° culture conditions and genetic methods were used [25]. *ifa-2(rh85* and *nc16)*, *ifa-2*::*gfp*, and *erIs1* have been previously described [7,26]. CZ3464 (*juIs176* [IFB-1A::GFP + *rol-6(su1006)*]) was provided by the Caenorhabditis Genetics Center.

### Generation of mutant forms of IFA-2::GFP

**Fig 1:**
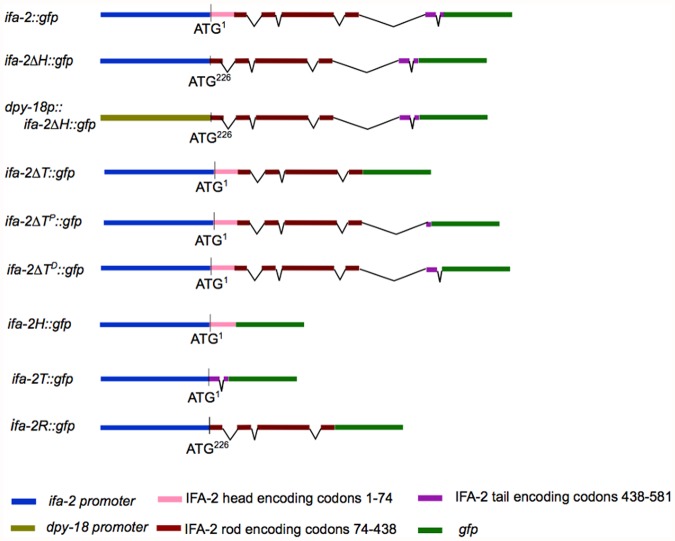
Overview of the variant *ifa-2*::*gfp* reporter genes used in this study. Specific arrays used in the study were the integrated *erIs1*[*ifa-2*::*gfp*] and the extrachomosomal *erEx7*[*dpy-18p*::*ifa-2* ^*∆H*^::*gfp;* pRF4]; *erEx9*[*ifa-2* ^*∆T*^::*gfp;* pRF4]; *erEx15*[*ifa-2* ^*∆TD*^::*gfp; pRF4*]; *erEx17*[*ifa-2R*::*gfp; pRF4*]; *erEx18*[*ifa-2R*::*gfp; pRF4*]; *erEx19*[*ifa-2R*::*gfp; pRF4*]

The *ifa-2* ^*∆H*^::*gfp* construct (Fig. 1) was generated by PCR amplification of *ifa-2*::*gfp* plasmid sequence into two partially overlapping fragments of DNA. The primers for fragment A were: U1721: 5′ CGAGACGCGTATCATGGAGCTCAATGATCGT 3′ and L197 5′ CCCCCAAAAAGCAAAAGCAGGAAA 3′ and for fragment B: U90: 5′ TTTCTTCGCACGTCTGGGCCTCTC 3′ and L1532: 5′ CTCGACGCGTGTCTGAAAATTTTAAATTC 3′. The primers that flanking the region to be deleted had Mlu1 restriction enzyme sites added at their 5′ ends. Amplification using Vent polymerase (NEB, Ipswich MA) were 93° for 3 minutes, followed by 15 sec at 93°, 30 sec at 50° and 10 min at 68° for 35 cycles. The resulting fragments were purified, digested with BmgB1 and Mlu1 for 2 hrs at 37°, and then ligated overnight at 16°. Despite the ability of this construct to rescue *nc16* (see results) GFP fluorescence associated with it was very low, and a second *ifa-2* ^*∆H*^::*gfp* derivative, *dpy-18p*::*ifa-2* ^*∆H*^::*gfp*, in which the native *ifa-2* promoter was replaced with the epidermis specific *dpy-18* promoter was generated by exchanging the *ifa-2* promoter region with the *dpy-18* promoter region of pBHDpy-18::gfp2 (kindly provided by Steve L’Hernaut, Emory University). An ~3kb fragment (fragment C) containing the *dpy-18* promoter sequence was amplified from pBHDpy-18::gfp with primer U90 and primer dpy18rev (5′ CTCGGACGCGTGTCTGAAAATAACTTCCTTAT 3′) containing an engineered Mlu I site at 5′ end. Fragment C was digested with BmgB1 and Mlu1 and subsequently ligated to BmgB1 and Mlu1 treated fragment A overnight to create the *dpy-18p*::*ifa-2ΔH*::*gfp* construct.

The *ifa-2* ^*∆T*^::*gfp* was generated by PCR amplification of a single fragment from *ifa-2*::*gfp* plasmid sequence containing all IFA-2::GFP sequences except the tail domain. The primers used were GGACGCGTGATAAGATGTTCTGCTGGAGTGTTCT and GGACGCGTTCCAACGTCAAAGCCAACAAACGACA; each contained an engineered Mlu-1 site. Amplification using Phusion mix (NEB, Ipswich MA) was for 98° for 30 seconds, then 25 cycles of 5 sec at 98°, 10 sec at 67° and 3.5 min at 72°. The resulting 7 kb amplified fragment was gel purified, Mlu1 digested for 1 hr at 37°, heated to 65° for 20 minutes and self-ligated using NEB Quick Ligase (NEB, Ipswich, MA) for 10 minutes at room temperature. The ligated DNA was transformed directly into competent JM109 cells.

To generate the proximal tail deletion, a similar protocol was used with the primers being 5′ GGA CGC GTT TAT TAG TGA CTA CAG TCA ATG G 3′ (3F) and 5′ GGA CGC GTG TGT CGA ATT GAT AAT CTA ATG C 3′ (3R). The distal tail deletion was generated by amplifying two partially overlapping fragments from the *ifa-2*::*gfp* plasmid that were then ligated to form *ifa-2* ^*∆Td*^::*gfp*. Primers 4F and 4R were synthesized with a 5′ Mlu1 restriction site and 1B and 2A were synthesized with a 5′ Sph1 restriction site for future ligation (IDT, Coralville IA). The primers were: 5′ GGA CGC GTC AAA AAT GAC CAA ACA TCT TG 3′ (4F) and 5′ CTGAAATCACTCACAACGATGGATAC 3′ (1B). The primers for the second fragment were 5′ CCTTTACAACTGCTGCAGGCATGCAA 3′ (2A) and 5′ GGA CGC GTT AAG GGA GAG GTA ATT CAA C 3′ (4R).

The *ifa-2H*::*gfp* was generated by PCR amplification of a single fragment from *ifa-2*::*gfp* plasmid sequence containing only the IFA-2::GFP promoter and head domain sequences using primers HOFor1 (GGACGCGTTGGAGGGTACCGGTAGAAAAAATGA) and HORev1(GGACGCGTTTCTTTCTTTTCACGCTCACGGTT) as described above for *ifa-2∆T*::*gfp*. Likewise the *ifa-2T*::*gfp* was generated by PCR amplification of a single fragment from *ifa-2*::*gfp* plasmid sequence containing only the IFA-2::GFP promoter and tail domain sequences using primers TOFor2 (GGACGCGTAATGAAGAGGCTGACACCGAA) and TORev2

(GGACGCGTGGAATCTGGATCGGTCATTATGTCT). The ifa-2R::gfp, rod only construct was generated from the *ifa-2∆H*::*gfp* plasmid sequence by PCR amplification of a single fragment of DNA containing all IFA-2∆H::GFP sequences except the tail domain using primers GGGATATCTCCAACGTCAAAGCCAACAAACGACA and GGGATATCGATAAGATGTTCTGCTGGAGTGTTCT. All constructs were sequenced to confirm the alterations.

To generate an RFP tagged IFA-2, the *ifa-2* portion of full length *ifa-2*::*gfp* was amplified using primers GFPrepR GGACGCGTCATTTTTTCTACCGGTACCCTCC and GFPrepIF CCGATATCTAGCATTCGTAGAATTCCAACTG. The *rfp* was amplified from a plasmid containing tagRFP inserted in pPD117–01 (gift of Robert Steven, University of Toledo) using primers RFPinsF (MluI) GGACGCGTAGTGTGTCTAAGGGCGAAG and RFPinsR (EcoRV) CCGATATCCTTGTATGGCCGGCTAGC. The amplified fragments were digested with Mlu1 and EcoRV, ligated, and the resulting *ifa-2*::*rfp* construct (pKW41) verified by sequence.

### Transgenic lines

DNA containing individual *ifa-2*::*gfp* variant DNA along with the marker plasmid *pRF4* was germline microinjected into N2 animals using standard protocols [27]. Roller progeny of injected animals were picked onto individual plates and those that produced a high proportion of F2 Rollers selected as stable transgenic lines. Because transgenic animals carried the variants in extra-chromosomal arrays, mosaicism and variation in expression levels between individuals carrying the same array might be expected. To minimize this issue we selected for use only the most stable lines (i.e. those that showed the highest transmission frequencies). We chose not to generate integrated lines for this work, since this can introduce additional mutations into the transgenic animals.

IFA-2::GFP variant transgenes were introduced into *ifa-2(nc16)* animals by standard genetics crosses. To generate IFA-2::RFP, IFB-1::GFP double transgenic animals, pKW41 carrying males were crossed with CZ3464 hermaphrodites, and F1 animals expressing both RFP and GFP selected to establish double transgenic lines. To examine localization of the fluorescent proteins, potential array-bearing animals were mounted in a drop of M9 buffer on a 1% agar pad slide and cover-slipped. Slides were examined under epifluorescence on a Zeiss Axiophot microscope, and photos obtained using either a Spot (Diagnostic Instruments, Inc., Sterling Heights, MI) or QIcam (QImaging, Surrey BC) camera, and processed with either Adobe Photoshop software running on a G4 Macintosh or Q Capture Pro software on a Dell Optiplex running Windows XP SP3.

## Results

**Fig 2:**
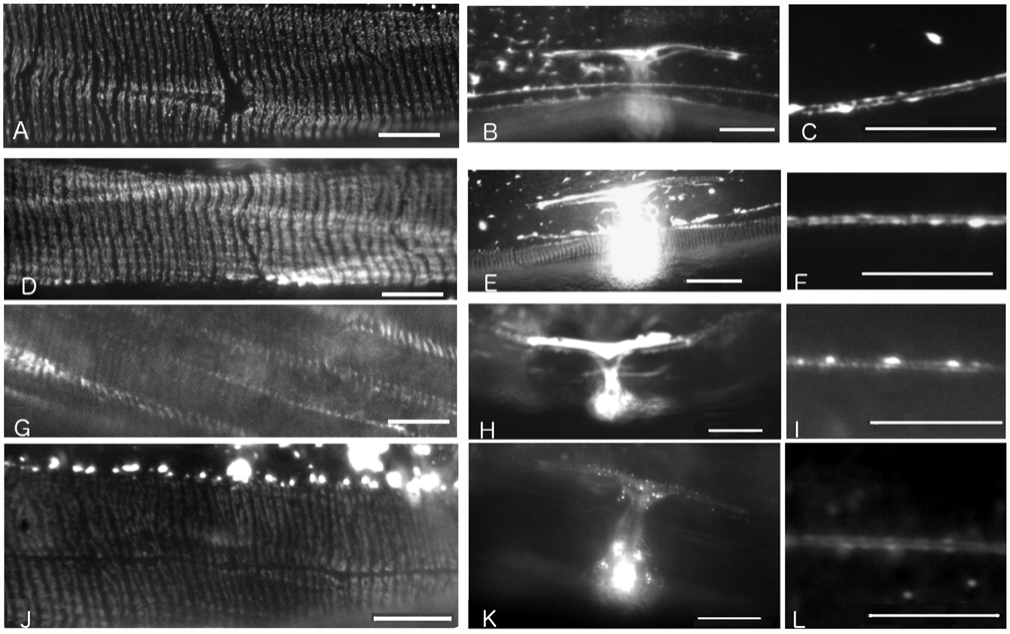
Fluorescence micrographs showing IFA-2::GFP variant localization in live adult transgene carrying lines. Scale bars for FO and ALM micrographs are 10μm, for uterus micrographs the scale bars are 20 μm. A) Full length IFA-2::GFP fusion protein localizes to epidermal FOs in rescued *ifa-2*::*gfp; ifa-2(nc16)* animals. The GFP-dependent fluorescence is detected in thin stripes oriented perpendicular to the long axis of the worm in regions of the epidermis adjacent to muscle. B) Full length IFA-2::GFP fusion protein localizes to uterine seam and to C) ALM in *ifa-2*::*gfp; ifa-2(nc16)*. D, E, F) Localization of headless IFA-2^*∆*H^::GFP protein is indistinguishable from IFA-2::GFP. Genotype shown is *ifa-2* ^*∆H*^::*gfp; ifa-2(nc16)* G,H, I) Localization of IFA-2^∆T^::GFP protein is indistinguishable from IFA-2::GFP. Genotype shown is *ifa-2* ^*∆T*^::*gfp; ifa-2(+)*. J,K,L) Localization of IFA-2^R^::GFP protein is indistinguishable from IFA-2::GFP. Genotype shown is *ifa-2* ^*R*^::*gfp; ifa-2(+)*.

An *ifa-2*::*gfp* fusion gene that completely rescues the *ifa-2* null mutation *nc16* had been previously generated (Table 1; also see reference [7]). In these rescued *ifa-2(nc16); erIs1* transgenic animals, IFA-2::GFP protein localizes to the epidermal FOs, the uterine seam and touch neurons (Fig. 2, panels A-C; also see reference [7]). The seam expression appears at the L4-adult molt and probably correlates with expression of IFA-2 in both the utse and the seam cells as they terminally differentiate (see below and discussion). To determine if the head domain of IFA-2 is essential for its localization and function, an *ifa-2*::*gfp* gene deleted for the head domain (*ifa-2* ^*∆H*^::*gfp*) was engineered *in vitro*, and reintroduced into wild-type and *nc16* null animals (Fig. 1). Translation of the *ifa-2* ^*∆H*^::*gfp* mRNA is predicted to initiate from the AUG at nucleotide position 226 (corresponding to M79 in the full length protein), resulting in an IFA-2::GFP protein containing only the rod and tail domains. Surprisingly, the *ifa-2* ^*∆H*^::*gfp* transgene was able to completely rescue the *nc16* null mutation, and has no dominant-negative effects when expressed in *ifa-2(+)* animals (Tables 1 and 2). In rescued animals, the IFA-2^ΔH^::GFP protein localizes to FOs, touch neurons, and the uterine seam in a pattern indistinguishable from wild-type IFA-2::GFP (Fig. 2, panels D-F). These results demonstrate that a headless IFA-2 protein can localize normally and that the head domain is not essential for IFA-2 specific function. One possibility is that the head domains of other cytoplasmic IFs associated with the FO serve a redundant function and are able to compensate for loss of the IFA-2 head.

**Table 1:** Rescue of *ifa-2* mutants by transgenes carrying IFA-2::GFP deletion variants.

Genotype of parent	IFA-2 variant in array	Percent of array containing progeny of the indicated phenotype (n)		Percent of progeny w/o array of the indicated phenotype (n)	
		Non-Mua	Mua	Non-Mua	Mua
+/+	no array	*	*	100 (102)	0
*nc16; erIs1*	ifa-2	100 (95)	0	*	*
*nc16; erEx7*	ifa-2∆H	96 (79)	4 (3)	0	100 (44)
*nc16/+; erEx15*	ifa-2∆TD	73 (127)**	27 (47)	76 (157)**	24 (50)
*nc16/+; erEx19*	ifa-2R	72 (85)**	28 (33)	76 (99)**	24 (32)

* genotypic category not present in progeny

** includes nc16/+ and +/+ progeny

**Table 2:** Frequency of dominant Mua phenotype in transgenic IFA-2::GFP variant lines.

Genotype (n)	IFA-2 variant in array	Percent of array containing progeny of the indicated phenotype (n)		
		non-Mua Adult	Mua Adult	Mua late larval
*+/+*	no array	100 (102)	0	0
+/+; *erEx7*	ifa-2∆H	100 (92)	0	0
+/+; *erEx9*	ifa-2∆T	100 (231)	0	2
+/+; erEx15	ifa-2∆T^D^	93 (191)	3 (6)	4 (5)
+/+; *erEx19*	ifa-2R	93 (194)	7 (15)	0 (8)
+/+; *erEx17*	ifa-2H	88 (167)	0	12 (23)
+/+; *erEx18*	ifa-2T	100 (89)	0	0

**Fig 3:**
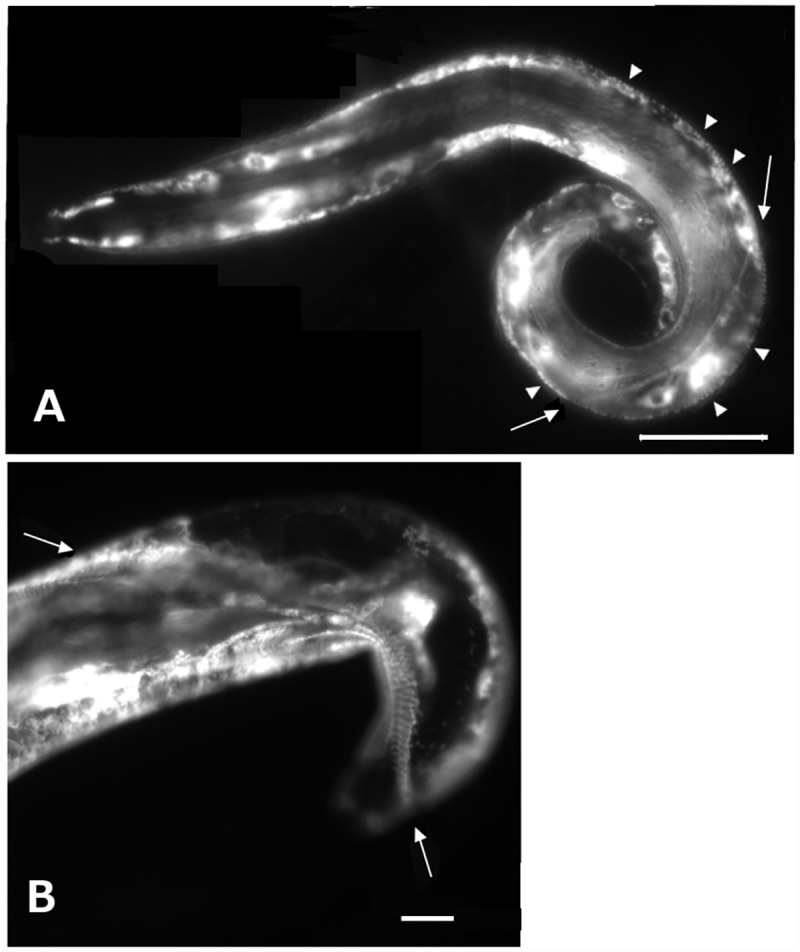
Fluorescence micrographs of animals displaying a dominant-negative phenotype associated expression of *ifa-2*::*gfp* variant. A) Fluorescence micrograph of a representative *ifa-2* ^*∆T*^::*gfp* animal displaying a dominant negative phenotype in a wild-type background. Expression is apparent in the epidermal cells and incorporation of IFA-2^∆T^::GFP into FOs visible (arrowheads). A region of epidermal separation associated with collapsed muscle can be seen by the presence of displaced GFP decorated membrane (arrows). Scale bar is 25 μm. B) Photomicrograph of an animal expressing IFA-2H::GFP has muscle detachment, likely due to overexpression of the construct. Muscle displaced from the cuticle (arrows). Scale bar is 20μm.

In contrast, the IFA-2 tail domain is essential. A *ifa-2* ^*∆T*^::*gfp* transgene lacking amino acids 438–581 was not able to rescue animals homozygous for the null allele. This was not due to an inability to be expressed or localize to the FOs since IFA-2^∆T^::GFP localized to FOs in a pattern essentially indistinguishable from wild (Fig. 2, panels G-I). In an *ifa-2(+)* background, 1% of the animals expressing IFA-2^∆T^::GFP showed a dominant-negative Mua phenotype (Table 2, Fig. 3A).

In an attempt to further delineate the essential region of the tail domain in IFA-2 function, IFA-2::GFP variants deleted for either amino acids 478–532 (IFA-2^∆Tp^::GFP) or 534–572 (IFA-2^∆Td^::GFP) were generated. Both these variants localized to the FOs and were capable of generating dominant negative phenotypes. We could obtain F1 progeny of injected worms that expressed *ifa-2* ^*∆Tp*^::*gfp* in an *ifa-2(+)* background with phenotypes ranging from Mua larvae to healthy adults, however we were unable to generate a transmissible line to test whether IFA-2^∆Tp^::GFP could rescue mutants. Similar variable dominant negative phenotypes were seen in the *ifa-2∆* ^*Td*^::*gfp* transgenic lines, and this variant was unable to rescue the *ifa-2* null allele (Tables 1,2). These results suggest that the essential portion of the tail domain either spans amino acid position 533 of the tail, or it is comprised of parts of both regions.

To determine if the rod alone was sufficient to localize IFA-2 to FOs, an IFA-2 variant containing only amino acids 74–438 of the rod domain fused to GFP was generated (IFA-2R::GFP). In otherwise wild-type animals carrying the *ifa-2R*::*gfp* transgene, IFA-2R was visibly seen to localize to FOs in 87% (27/31) of the larval and 62% (60/97) of the adult animals (Fig. 2, panels J-L, Table 3). The FO associated fluorescence in many of these animals appeared fainter than seen in the case of intact IFA-2::GFP, and it is possible that some of the animals scored as negative still had minimal incorporation. In 2 of the 60 FO positive adults, the patterning was patchy. In all the transgenic animals, across multiple lines, strong localization of fluorescence to the uterine seam (in adults) and fibrous or punctate fluorescent accumulations in the epidermal ridges were observed. These results suggest that although the head and tail domains may contribute to assembly of IFA-2 into mature filaments and their incorporation into FOs, the rod domain alone is sufficient. However, *ifa-2*::*gfp* was unable to rescue the *ifa-2* mutant (Table 1), showing that the rod alone lacks essential components of IFA-2 functionality.

**Table 3:** Head and rod but not tail drive GFP localization to the FOs.

Genotype	IFA-2 variant in array	% array carrying Larvae with GFP localizing to (n)		% array carrying Adults with GFP localizing to (n)		
		FO	Epidermis	FO	Epidermis	Utse
*+/+; erEx19*	ifa-2R	87(31)	100(31)	62(97)	98(97)	100(41)
*+/+; erEx17*	ifa-2H	69 (65)	100(65)	26 (39)	43(39)	74(39)
*+/+; erEx18*	ifa-2T	0 (66)	100(66)	0 (28)	100(28)	60(23)

**Fig 4:**
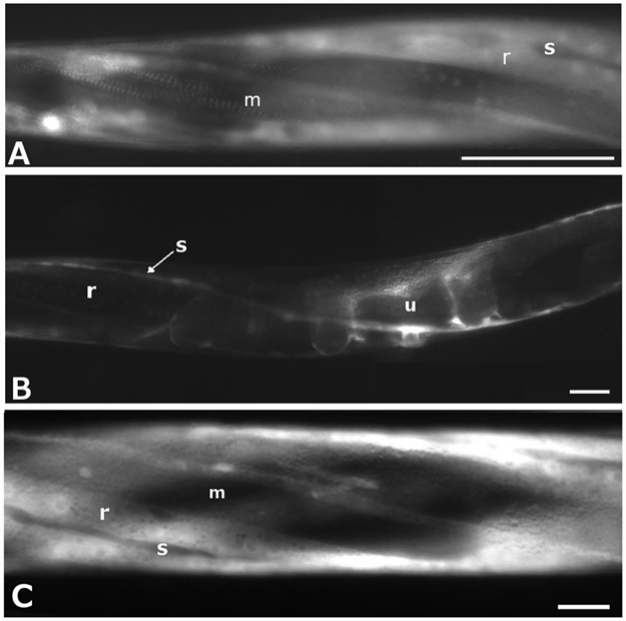
Fluorescence micrographs of representative animals expressing rod-deleted IFA-2::GFP variants in wild-type genetic background. Scale bars are 25μm. A) L4 stage with IFA-2H::GFP expressed in the epidermal ridges (r) and incorporation into FOs in the areas within the epidermis overlying muscle (m) is visible. Seam cells (s) do not express the tagged protein. B) Adult with IFA-2H::GFP expressed in the seam (s), expression in epidermal ridges (r) is not obvious. Uterine expression (u) includes both utse and uv cells. C) L4 stage with IFA-2T::GFP expressed at high levels in the epidermal ridges (r) but not seam cells (s). Incorporation into FOs in the areas of epidermis overlying muscle (m) is not detectable.

To test whether the head or tail domains could interact with and bind FOs in the absence of a rod domain, GFP-tagged constructs that included only the head (amino acids 1–74; designated IFA-2H::GFP), or the tail (amino acids from 438–581; designated IFA-2T::GFP) of IFA-2 fused to GFP were expressed. IFA-2H::GFP was observed in the FOs of 69% (45/65) of larval stage transgenic animals, but only 26% (10/39) of the adults (Table 3), often as patches rather than continuously within the band (Fig. 4A). Diffuse expression in the epidermal ridges was observed in all larvae, but was undetectable in 56% of the adult animals. Expression was absent from the epidermal seam cells in larvae, but was observed in 38 of 39 adults, suggesting that IFA-2 expression is normally initiated in seam cells after they terminally differentiate (Fig. 4B). In 12% of larvae carrying the transgene a dominant-negative Mua phenotype was observed (Fig. 3B, Table 2). The Mua phenotype correlated with higher levels of transgene localization to the FOs as 95% (19/20) of rolling (i.e array carrying) Mua animals had qualitatively bright expression compared to 22% (5/23) of rolling non-Mua animals at similar stages that had lower intensities of incorporation at the FO. Both the localization and the dominant negative phenotype are consistent with the head domain directly interacting with proteins in the FO.

The tail-only construct, IFA-2T::GFP, did not localize to the FOs at any developmental stage (0/66 larvae; 0/28 adults, Table 3). Diffuse fluorescence was present in the epidermal ridges in all the animals, but FOs incorporation was never observed, nor was a dominant negative phenotype, suggesting that any interactions between the tail domain and proteins at the FO requires other IF domains to direct it to the FO. Thus, although the tail domain is necessary for normal IFA-2 function, its localization and interaction with proteins at the FOs appears dependent on the rod domain.

**Fig 5:**
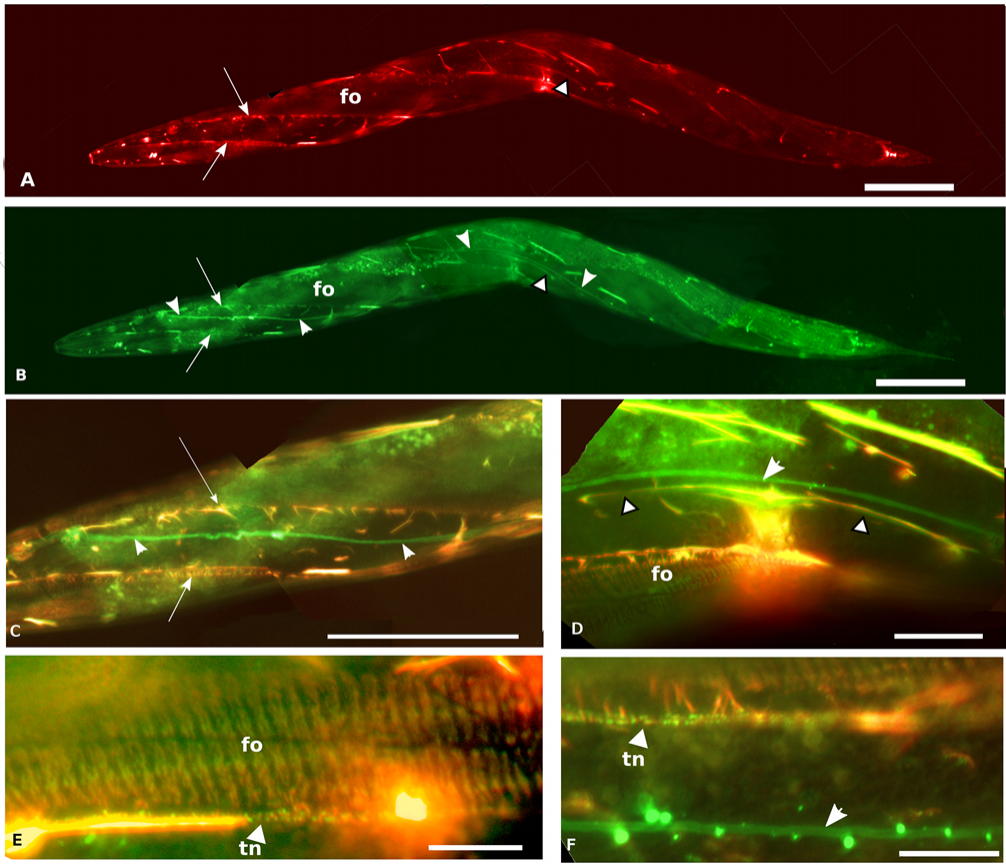
Fluorescence micrographs of adults co-expressing IFA-2::RFP and IFB-1::GFP. Scale bars are 50 μm in panels A-C, 10 μm in panels D-E. A) IFA-2::RFP is expressed in fibrous organelles (fo), uterine seam (arrow-head with black outline). Arrows indicate a region of brighter fluorescence that coincides with the edge of the muscle quadrant, the lower line of bright fluorescence also includes fluorescence associated with the touch neuron. B) Same worm as in A, showing IFB-1::GFP expression. Note co-incidence of expression with IFA-2::GFP in fibrous organelles (fo), and uterine seam (arrow-head with black outline). Excretory canal shows IFB-1 expression (all-white arrowheads) that is not associated with IFA-2 co-expression. Punctuate auto-fluorescence of gut granules is also observable in this panel. Ectopic accumulations of IFB-1 in the epidermal ridges co-localize with accumulations of IFA-2::RFP at the same locations. C) Enlargement and red/green overlay of a portion of anterior body showing co-incidence of IFA-2::RFP and IFB-1::GFP expression. Arrows and arrowheads identify the same structures as seen in A and B. Both the co-localization of IFA-2 and IFB-1 in the touch-neuron (adjacent to lower arrow) and in ectopic accumulations is apparent. (The punctate pattern of the FOs is not in the plane of focus of this image.) D) Enlargement and red/green overlay of uterine seam region showing IFA-2::RFP and IFB-1::GFP expression. Co-localization of IFA-2 and IFB-1 in the uterine seam (arrow-heads with black outline), FOs (fo) and in ectopic accumulations is readily apparent. The IFB-1-only expressing excretory canal is indicated with the all-white arrowhead. E) 1,000x red/green overlay photomicrograph of FOs (fo) and adjacent touch neuron (arrowhead labeled tn) showing co-expression of IFA-2::RFP and IFB-1::GFP expression. F) 1,000x red/green overlay photomicrograph of touch neuron (arrowhead labeled tn) showing co-expression of IFA-2::RFP and IFB-1::GFP, and excretory canal (arrowhead) that lacks IFA-2 expression.

Given the similar patterns of expression and the *in vitro* observation that IFA-2 can form heterodimers with IFB-1 [13], we wanted to determine the degree of localization overlap between IFA-2 and IFB-1 *in vivo*. Animals expressing both a IFA-2::RFP and IFB-1::GFP were generated using standard genetic methods (Fig. 5). All tissues with IFA-2::GFP expression also show IFB-1 co-expression, however expression of IFB-1 is observed in tissues that do not express IFA-2; presumably in these tissues IFB-1 dimerizes with an alternate partner. Ectopic accumulations of IFA-2 also contain IFB-1, suggesting that IFA-2′s incorporation into filaments requires dimerization with IFB-1.

## Discussion

Full length IFA-2 fused to GFP has previously been shown to functionally substitute for wild-type IFA-2 in homozygous *ifa-2* null worms [7]. The IFA-2::GFP protein localized to the FOs, uterine seam, and touch neurons in a pattern comparable to the MH4 antibody that recognizes several epidermal IFs, including IFA-2 [7,15]. Some ectopic accumulations of fluorescence in the epidermal ridges was also observed in IFA-2::GFP transgenic worms, and is likely due to excess, non-stoichiometric IFA-2::GFP expression from the fusion gene. Similar ectopic accumulations have been observed in IFB-1::GFP transgenic animals [8]. However, there was no obvious novel or dominant-negative phenotype associated with IFA-2::GFP expression. These observations encouraged us to probe the functional roles of specific IFA-2 domains by deleting them in IFA-2::GFP and comparing the functionality and localization of the deletion mutants to full length IFA-2::GFP. In addition, since the phenotype of the *ifa-2* null is distinct from that of *ifb-1*, its heteromeric partner [7,8], alterations that disrupt IFA-2 specific functions should be distinguishable from those that affect IF assembly or function more generally. In our studies, the IFA-2::GFP variants were expressed from extra-chromosomal arrays, somatic loss of the array resulting in mosaicism can impact variation in expression levels between animals and/or tissues within a single animal. However, the main epidermal tissue, hyp-7, is syncytial and, in principal, expression from any of the nuclei should be sufficient to provide gene product to the entire syncytium. Conversely, the variation in expression level provides a tool to look at the effects of varying levels of expression (as assayed by fluorescence intensity), on animal phenotype (e.g. potential dominant negative effects in IFA-2H::GFP carrying animals).

IFA-2::GFP lacking the head, tail, or both domains still localize to the FOs, uterine seam and touch neurons (see summary Table 4). IFA-2 normally forms heterodimers with IFB-1 *in vitro* [13], and we show that IFA-2 co-localizes with IFB-1 *in vivo* (Fig. 5) suggesting that localization to filaments requires an interaction with IFB-1. Presumably, the truncated IFA-2^∆H^::GFP, IFA-2^∆T^::GFP and IFA-2R::GFP proteins also dimerize with IFB-1 *in vivo*, forming heterodimers that assemble into mature filaments and are incorporated into the FOs. The alternative, that the truncated variants are directly binding the FOs without first assembling into filaments, seems unlikely for a number of reasons. First, IFA-2^∆H^::GFP can functionally substitute for native IFA-2, as assayed by null mutant rescue and it does not interfere with wild-type IFA-2 when expressed in an *ifa-2+* background, consistent with its assembly into dimers and filaments and arguing against significant direct FO binding. Secondly, the tail domain fused to GFP does not direct GFP to the FOs. Finally, although the head domain appears to have the ability to bind to FOs in the absence of the rod domain, this localization is incompletely penetrant, unlike the robust FO localization seen in the variant retaining both the head and rod domains.

**Table 4:** IFA-2 variants: phenotypic summary.

Variant	Rescues null allele	Localizes to FOs *	Localizes to uterine seam	Dominant negative phenotype
IFA-2::GFP	yes	+++	+++	not observed
IFA-2∆H::GFP	yes	+++	+++	not observed
IFA-2∆T::GFP	no	+++	+++	Mua
IFA-2R::GFP	no	++	+++	Mua
IFA-2H::GFP	**	+	++	Mua
IFA-2T::GFP	**	no	++	not observed

* +++ over 90% of animals show localization, ++ between 50% and 90% show localization, + less than 50% show localization

** not tested

Studies in vertebrate systems showing that IF proteins that lack a head or tail domain, but retain an intact rod domain, can assemble into filaments in the presence of an intact dimerization partner are consistent with the observation that neither the head nor tail domain of IFA-2 appears absolutely necessary for its assembly into filaments. For example, headless K14 can assemble into filaments containing intact K5, its natural heterodimeric partner [28], and tail-less K8 can form filaments with tail-less K18 or K19 [29] Headless vimentin can assemble into homopolymeric filaments in the presence of wild-type vimentin, although not in its absence, [30,31]and tail truncated variants of vimentin are able to assemble into filaments *in vitro* [31]. Finally, it is well established that direct interactions between the rod domains of individual IF proteins are the main driver of their assembly into dimers, tetramers and mature filaments [2,32]. Despite the ability of headless and tailless IFA-2 to localize to the FOs, a role for the IFA-2 head or tail domains contributing to the filament assembly is not precluded; they could play important but redundant roles in modulating the assembly and maturation of IFs. For example, head and tail domains have been suggested to function antagonistically in IF assembly with the head promoting assembly and the tail terminating assembly, at precisely 10nm filament stagger [33].

Given the exposed position of the IF head and tail domains in the mature filament, their primary role is likely to involve in direct interactions between the IFs and the FOs, as well as other cellular partners. In addition, the predicted phosphorylation sites on IFs are clustered in the head and tail domains, suggesting a role for regulation of IF interactions by posttranslational modifications at these sites [11]. This may include modulation of filament assembly and turnover. The IFA-2 tail domain, although not essential for IFA-2′s localization to the FOs, appears to be essential for IFA-2 function. IFA-2::GFP proteins that lack all or part of the tail domain still localize to the FOs, but are unable to rescue the null mutant, and result in a weak, partially penetrant dominant-negative Mua phenotype in an *ifa-2* ^*+*^ background (Table 4). Together, the dominant negative phenotype and FO localization suggest that IFA-2^∆T^::GFP is incorporated into filaments and as it replaces an increasing portion of native IFA-2, the result is an increasing loss of IFA-2 tail mediated function. In other systems, the tail domains of IF proteins have been shown to modulate assembly, maturation and the mechanical properties of filaments. Tail truncated desmin and vimentin, although able to assemble into filaments *in vitro*, form filaments with abnormal variations in width or reduced biophysical integrity [31,34]. Tailless K14 assembles with its heteromeric partner K15 *in vitro*, but bundling of the keratin filaments is impaired, with resulting weakening of the IFs [35]. In *C*. *elegans*, SUMOylation of the IFB-1 tail domain serves to maintain a cytoplasmic pool of IFs for exchange with filaments at the FOs, critical for turnover and remodeling of the FOs in response to stress as the worms grow in size [12]. The observed IFA-2^∆T^::GFP phenotypes are consistent the IFA-2 tail domain contributing in some similar fashion. It is unlikely, however, that the IFA-2 is involved in initial filament assembly competence or in FO incorporation, since tail truncated IFA-2::GFP localizes to the FOs even in *ifa-2* null animals. More likely, the IFA-2 tail interacts with FO-associated proteins to mediate an IFA-2 specific function. This function does not appear to be an ability of the tail to direct or bind IFs to the FOs, since IFA-T::GFP did not localize to FOs at any stage of development, despite clear epidermal expression. This contrasts with the reported ability of the K14 tail to localize to keratin filaments *in vivo* [35] and the mislocalization of complete K8/K18 or K8/K19 filaments in which neither partner had a tail [29]. Furthermore, IFA-2T::GFP did not interfere with native IFA-2 function, as dominant negative effects were never observed, again suggesting an inability of a tail domain to robustly interact with potential partners at the FO unless tethered to the filaments via a rod domain.

In contrast, the IFA-2 head domain is not essential for IFA-2 function. This likely reflects a redundancy of function between the IFA-2 head and one of the other epidermally expressed IFs. The observations that headless IFA-2^∆H^::GFP protein can functionally substitute for native IFA-2, while IFA-2H::GFP that contains only the head domain interferes with epidermal IF function in a dominant negative manner, is consistent with this. Since the IFA-2 head appears to have an intrinsic ability to bind to the FOs we suggest that head domain of IFA-2 as well as those of IFB-1, IFA-3 and/or IFC-1 bind to FO associated proteins. One simple model is that the IF head of one of the other epidermally expressed IFs has the same function and binds the same partners as the IFA-2 head. The observed dominant negative phenotype of IFA-2H::GFP would result from it blocking interaction sites common to both IFA-2 and one of the other IF heads. IFA-3 is a strong candidate, 66 of the 75 amino acids positions in the head domains of IFA-2 and IFA-3 are identical, 8 of the remaining 9 are conservative changes, and both proteins form heterodimers with IFB-1. Thus, if the heads of both IFA-2 and IFA-3 play redundant roles, and they bind similar partners in the FO, IFA-3 would be able to compensate for loss of the head in IFA-2^∆H^ animals. Overlap of head domain function has been experimentally observed in vertebrates; even the widely diverged head domain of a vertebrate lamin is able to substitute for the head of a neurofilament in IF assembly [36].

Although IFA-2 head function appears to be redundant, its ability to localize to the FOs in the absence of the rod and tail, suggest that it may be important in localizing IFs to the FOs, or conversely localizing hemidesmosome proteins to the FO. In other organisms, IF head domains have been implicated in both modulation of filament assembly and in filament localization. Phosphorylation of the desmin head inhibits its assembly into filaments; phosphorylation of the neurofilament NF-L head modulates its axonal transport [37,38]. The head domain of vimentin interacts with proteins exerting a nuclear directed force [39] and the head domains of type II keratins associate with the “cell envelope”, possibly involving its direct binding to desmosplakin [40]. In vertebrate neuronal IFs, the head domain specifies the heterodimeric partner of assembly; the specific mechanism has yet to be identified [41]. Finally the head domain of GFAP associates with 14–3–3 proteins when phosphorylated, and this interaction may contribute to the regulatory dynamics of GFAP containing filaments in vivo [42].

During the L4-adult molt significant remodeling of the junctions between the uterus and epidermis occurs, including formation of the uterine-vulval connection and mechanical connections of the utse with the overlying seam cells [43]. As part of his process the seam cells terminally differentiate and fuse to form a syncytium. IFA-2 and IFB-1 become expressed in the uterine seam, however expression remains localized to this area of connection [7][8]. It had been shown that IFA-2 is expressed in the utse [7], but it was not clear whether there was also expression of IFA-2 in the seam cells that connect to utse at the uterine seam. The observation of GFP fluorescence in the seam syncytium distal to the uterine seam in IFA-2H::GFP and occasionally IFA-2T::GFP adults suggests that IFA-2 is also expressed in the seam cells after the larval to adult switch. The IFA-2H::GFP and IFA-2T::GFP proteins are presumably able to diffuse throughout the seam syncytium, while full length IFA-2 and the other rod containing variants remain tethered to the seam, suggesting that assembled filaments are spatially restricted to the uterine seam itself.
